# ELM Meets Urban Big Data Analysis: Case Studies

**DOI:** 10.1155/2016/4970246

**Published:** 2016-08-29

**Authors:** Ningyu Zhang, Huajun Chen, Xi Chen, Jiaoyan Chen

**Affiliations:** ^1^College of Computer Science and Technology, Zhejiang University, Zhejiang 310014, China; ^2^Zhejiang University, Zhejiang, China

## Abstract

In the latest years, the rapid progress of urban computing has engendered big issues, which creates both opportunities and challenges. The heterogeneous and big volume of data and the big difference between physical and virtual worlds have resulted in lots of problems in quickly solving practical problems in urban computing. In this paper, we propose a general application framework of ELM for urban computing. We present several real case studies of the framework like smog-related health hazard prediction and optimal retain store placement. Experiments involving urban data in China show the efficiency, accuracy, and flexibility of our proposed framework.

## 1. Introduction

Urban computing is a process of acquisition, integration, and analysis of big and heterogeneous data generated by diverse sources in urban spaces, such as sensors, devices, vehicles, buildings, and humans, to tackle the major issues that cities face (e.g., air pollution, increased energy consumption, and traffic congestion) [1]. With the rapid progress of urban computing, lots of applications have appeared to analyze amounts of urban data using big data analysis technologies for real practical problems. Most of them use conventional machine learning and computational intelligence solutions. However, with the exponential growth of data and complexity of systems, those methods have suffered lots of bottlenecks in learning (e.g., intensive human intervention and convergence time).

In fact, the urban data acquired in real situations normally are heterogeneous and in big volumes. Thus, according to studies [2], treating features extracted from different data sources equally does not achieve the best performance. Moreover, most of the models of applications are hard to implement in real situations because of the complexity and sparsity of some data. Therefore, fast machine learning and computational intelligence techniques are needed.

Extreme learning machine (ELM) [3] can be a good solution for the learning of large sample data as it has high generalization performance and fast training speed. Moreover, the stacked ELMs [4] perform well when facing extremely large and complex training datasets. Indeed, ELM is flexible and easy to implement. In this paper, we propose a general application framework of ELM for urban computing.

To solve challenges in urban computing using ELM, we firstly analyze the types, sources, and structures of urban data. We standardize the form of data from different sources and fuse data across modalities as input of ELM. In fact, the urban data can be obtained from online social media and offline physical sensors. Online social media (twitter, user comments) have opinions about places. These data are normally text (user ratings are numeric) and uncertain. The bad situation is that the amounts of these data are influenced by the populations or type of district to a great extent. For instance, it is quite easy to retrieve amounts of data in a metropolis. However, less-developed regions have small populations and, hence, relatively low social media activity. Meanwhile, offline data reflect the physical condition of this region such as the flow of taxis and buses, traffic congestion index, real estate, POIs, road network, air quality, and meteorological elements. The amounts of these data in different regions are usually similar. Nevertheless, these data are from different sources and are heterogeneous. We divide the city into regions by the road network. We obtain huge amounts of data in each region. We formalize these data and build standard feature vectors including social view from online social media and physical view from physical sensors through location based feature selection. Social view and physical view are treated as different views on an object. We adopt deep autoencoder method [5] to capture the middle-level feature representation between two modalities (e.g., social view and physical view). Then, we train the data flexibly, which means that the data with different sparse degree will be trained by different kinds of ELM based methods.

Furthermore, we propose several case studies of our framework. For different real applications, we can easily add or remove data sources and adjust parameters of the model. We use the social media, meteorological elements, and air quality to make smog-related health hazard prediction. Truly, these data are heterogeneous, while stacked ELM [4] can flexibly train these data. We use user comments, social media, the flow of taxis and buses, traffic congestion index, real estate, POIs, and road network to help optimally retain store placement. In real situations, the user comments data suffer the data sparsity problem. We handle this by transferring knowledge from regions with huge data by domain adaptation transfer ELM [6].

The major contributions of this paper are as follows:

1. We propose a general application framework of ELM for urban computing.
2. We propose several case studies for our framework such as smog-related health hazard prediction and optimal retain store placement.
3. We evaluate our framework in real urban datasets and reveal the advantages in precision and learning speed.

The remainder of this paper is organized as follows.  Section 2 briefly reviews existing studies on ELM and urban computing.  Section 3 presents the framework and key challenges.  Section 4 describes the case studies.  Section 5 presents the results of our experiments. Finally, Section 6 summarizes our findings and concludes the paper with a brief discussion on the scope for future work.

## 2. Related Work

### 2.1. ELM

Extreme learning machine (ELM) has recently attracted many researchers' interest due to its very fast learning speed, good generalization ability, and ease of implementation [3]. Zhou et al. [4] proposed stacked ELMs (S-ELMs) which are specially designed for solving large and complex data problems. The S-ELMs serially connect stacked small ELMs divided by a single large ELM network and can approximate a very large ELM network with small memory requirement. Moreover, the S-ELMs support the ELM autoencoder during each iteration which significantly improves the accuracy on big data problems. Therefore, it has great chance to improve efficiency and precision using S-ELMs to train heterogeneous data. However, it is necessary to have a standard data modal for it.

With the rapid growth of cities, huge amounts of data can be obtained, while there still exist data sparsity problems as a result of sensor coverage, difference of human activities, and so on. L. Zhang and D. Zhang [6] proposed a unified framework, referred to as Domain Adaptation Extreme Learning Machine (DAELM), which learns a robust classifier by leveraging a limited number of labeled data from target domain for drift compensation as well as gases recognition in E-nose systems, without loss of the computational efficiency and learning ability of traditional ELM. Nevertheless, different urban data have different modalities and are difficult to transfer knowledge directly.

### 2.2. Cross-Domain Data Fusion

The data from different domains consist of multiple modalities. According to [7], there are three categories of methods that can fuse multiple datasets: (1) stage-based fusion methods [8], (2) feature-level based methods [5, 9], and (3) semantic meaning-based methods [10–12]. Ngiam et al. [5] proposed deep autoencoder method to capture the middle-level feature representation between two modalities (e.g., audio and video).

However, there is a lack of research which utilized methods to fuse both social media data and physical sensor data for a general application framework for urban computing. This is mainly due to the lack of (1) systematic approaches for collecting, modeling, and analyzing such information and (2) efficient machine learning framework which can combine features from both social and physical views.

### 2.3. Urban Computing

The urban data of a city (e.g., human mobility and the number of changes in a POI category) may indicate real conditions and trends in the cities [13].

For instance, the tweets from social media and meteorological elements may indicate the existence of smog-related health hazards. Chen et al. [14] modeled smog-related health hazards and smog severity through mining raw microblogging text and network information diffusion data and developed an artificial neural network- (ANN-) based model to forecast smog-related health hazard with the current health hazard and smog severity observations. This paper is a follow-up work and as social media and physical sensors are different modalities, we adopt urban knowledge fusion and propose a general framework.

Furthermore, human mobility with POIs may have contributions to the placement of some businesses. Scellato et al. [15] used the location based social network to study the optimal retail store problem. They collected and analyzed data from Foursquare to understand the popularity of the three major retail stores in New York. They evaluated a diverse set of data mining features and analyzed the spatial and semantic information about location and mode of user action in the surrounding area. The problem arises when we cannot obtain sufficient online data in some small cities. This paper is also follow-up work of [16].

## 3. Approach

### 3.1. Key Challenges

According to [1], urban computing has three main challenges: urban sensing and data acquisition, computing with heterogeneous data, and hybrid systems blending the physical and virtual worlds. In real situations, many conventional machine learning methods suffer key challenges summarized as follows.

*(1) Poor Learning Efficiency.* As there are huge urban data generated from social media and physical sensors, it is great challenge for the training efficiency of machine learning. Seriously, many applications of urban computing have timeliness requirements such as disaster and traffic monitor. It is very urgent to train a model as fast as possible and get the final result.

*(2) Complex Model to Handle Heterogeneous Data.* Data from different sources consist of multiple modalities, each of which has different representation, distribution, scale, and density. For example, text is usually represented as discrete sparse word count vectors, whereas an image is represented by pixel intensities or outputs of feature extractors which are real-valued and dense. POIs are represented by spatial points associated with a static category, whereas air quality is represented using a geotagged time series. Human mobility data is represented by trajectories, whereas a road network is denoted as a spatial graph. Treating different datasets equally or simply concatenating the features from disparate datasets cannot achieve a good performance in data mining tasks. As a result, fusing data across modalities becomes a new challenge in big data research, calling for advanced data fusion technology.

*(3) Sparsity of Data and Hard for Implementation.* It is quite easy to retrieve kinds of data in a metropolis. Nevertheless, some relatively small cities have small populations and, hence, relatively low social media activity. Specifically, we face the following two challenges: (1) the label scarcity problem and (2) the data insufficiency problem. Therefore, it is difficult to train and implement in real situations.

### 3.2. General Framework

**Figure 1:**
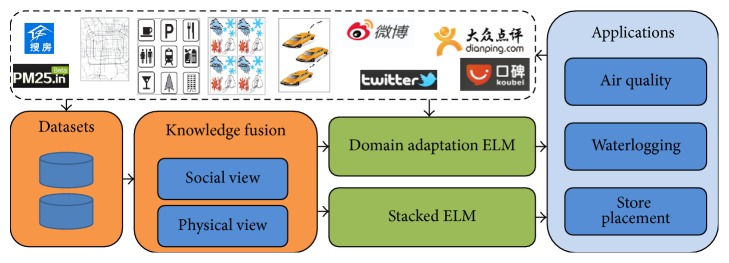
General application framework of ELM for urban computing.

As Figure 1 shows, our framework consists of two parts: urban knowledge fusion and training which is based on ELM. We firstly fuse the data obtained from both social media and physical sensors using deep autoencoder [5]. For those with abundant data, we use stacked ELM [4] to train. For those with sparse data, we use DAELM [6] and transfer knowledge from regions with abundant data.

### 3.3. Typical Technologies

#### 3.3.1. Map Segmentation

**Figure 2:**
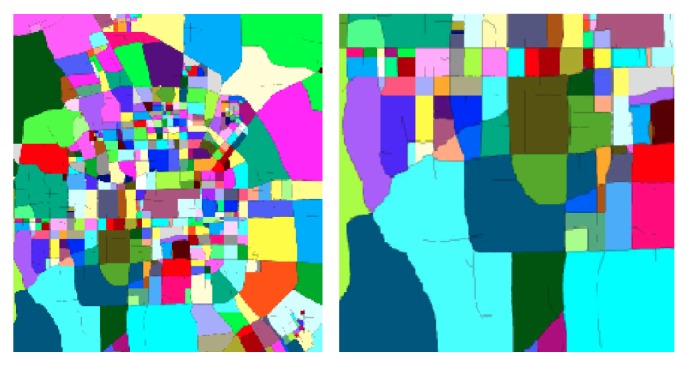
Segmented regions.

We divide a city into disjointed blocks (https://github.com/zxlzr/Segment-Maps), assuming that placement in a block *g* is uniform. Road network is usually composed of a number of major roads, such as the ring road; the city is divided into areas [13]. We map the projection of the vector-based road network on a plane. Then, the road network is converted into a raster model [17]. Actually, each pixel of a projected map image can be viewed as a block element of a raster map. Consequently, the road network is converted into a binary image. Then, we extract the skeleton of the road, while retaining the original two-value image topology.  Figure 2 shows the result of the procedure described above for Beijing's road network. Finally, we get the blocks *g* of cities.

### 3.4. Location Based Feature Selection

For each block, we can access the features over its neighbourhood and simply calculate the average value. However, there exist problems if ignoring the location information of the surrounding features. There are two reasons we need to consider. (1) From the distance perspective, if the selected location is far from the target location, it may have few impacts on the target location and vice versa. (2) From the density perspective, if one of the neighbour blocks has a lot of stores while the others have few, maybe this block has greater influence on the target location. So we propose a location based feature selection (LBFS) method to calculate features considering neighbourhood's impact. Suppose we want to calculate a feature of target block *g*; *g* has *t* neighbours *N*_1_, *N*_2_,…, *N*_*t*_, *f*_*i*_ is the feature vector of each block *N*_*i*_, *d*_*i*_ is the distance between *N*_*i*_ and *g*, *n*_*i*_ is the number of feature points (which means there are *n* stores in block *i*, and *m*_*i*_ is the measure of *N*_*i*_).

*(1) Distance Related Features.* Some kind of features may reduce the impact of the store's score with the increase of distance. So we weigh the features with distances. Formally, we have

(1) $$\begin{array}{c} f_{g1}=\sum \frac{f_{i}}{d_{i}}. \end{array}$$

*(2) Density Related Features.* Several features may have fewer impacts on the store's score with the increase of density of surrounding shops. So we weigh the features with density. Formally, we have

(2) $$\begin{array}{c} f_{g2}=\sum \frac{f_{i}}{n_{i}}. \end{array}$$

*(3) Measure Related Features.* There may exist some features that may have fewer impacts on the store's score with the increase of measure of surrounding regions. So we weigh the features with measure. Formally, we have

(3) $$\begin{array}{c} f_{g3}=\sum \frac{f_{i}}{m_{i}}. \end{array}$$

In the real situation, we use all three kinds of feature selection results. Normally each kind of feature in a grid has three final feature values as a vector. Formally, we have

(4) $$\begin{array}{c} f_{g}=LBFS\left(\;f,d,n,m\;\right), \end{array}$$

where *f* are the feature vectors of the neighbours, *d* are the distances between the middle of neighbours and middle of the target block, *n* are the numbers of feature points in neighbours, and *m* are the measures of neighbours.

#### 3.4.1. Urban Knowledge Fusion

**Figure 3:**
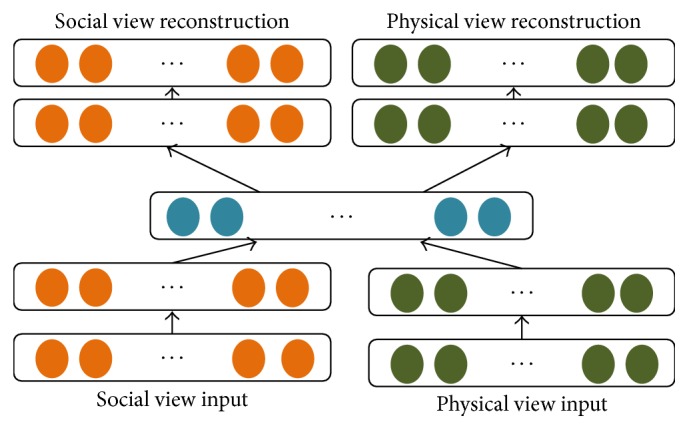
Deep autoencoder of social view and physical view.

For each block in cities we obtain the social view and physical view separately. Each view is represented as a feature vector. Social view is composed of social media text, user comment texts, user ratios, and so on according to different application requirements. Physical view is composed of physical sensor values like the flow of taxis and buses, traffic congestion index, real estate, POIs, road network, air quality, meteorological elements, and so on regarding different application requirements. We adopt the deep autoencoder [5] to capture the middle-level feature representation between social view and physical view. As Figure 3 depicts, deep learning effectively learns (1) a better single modality representation with the help of other modalities and (2) the shared representations capturing the correlations across multiple modalities.

#### 3.4.2. Training

Practically, we obtain the middle-level feature representation of each block. For those with abundant data, we use stacked ELM [4] to train. It can be used directly to solve regression, binary, and multiclass classification problems regarding different applications. For those with sparse data, we use DAELM [6] to transfer knowledge from regions with abundant data. Actually, we can treat different cities as different domains because data from different cities may have different distributions in feature and label spaces [18]. We use cities with abounded data as sources and transfer knowledge to target ones with sparse data.

## 4. Case Studies

### 4.1. Smog-Related Health Hazard Prediction

In fact, smog is a terrible health hazard that affect people's health according to recent research [19]. It is necessary to analyze, monitor, and forecast smog-related health hazards in a timely manner. In recent times, social media has become an increasingly important channel to observe sentiment, trends, and events. Furthermore, there are various physical sensors monitoring smog status, such as air quality stations, weather stations, and earth observation satellites, generating amounts of data about severity of smog. In this paper, we model the smog-related health hazards and smog severity by two indexes (PHI and SSI) using social media. The urban smog-related health hazard prediction problem is a classification problem to assign appropriate class labels (Public Health Index) to the blocks of cities.

Public Health Index (PHI) is the sum of total relative frequencies of smog-related health hazard phrases in the current tweets. D-PHI is an enhanced Public Health Index that includes consideration of diffusion in social networks.

*Definition.* Smog Severity Index (SSI) is the weighted sum of total relative frequencies of smog severity phrases in the current tweets. D-SSI is an enhanced Smog Severity Index that includes consideration of diffusion in social networks.

Firstly, we extract both smog-related health hazard phrases and smog severity phrases. Secondly, we gather raw tweets with time and location tags from Weibo (a twitter-like website in China). Thirdly, we calculate the daily relative frequency rf of each phrase:

(5) rfp=afp,TC×idfp,TH,afp,TC=∑d∈TCfp,dTC,idfp,TH=log⁡THd∈TH:p∈d,

where *T*_*H*_ and *T*_*C*_ represent historical and current tweet sets, respectively, *p* represents a phrase, *d* represents a tweet, f(*p*, *d*) represents the frequency of phrase *p* in tweet *d*, af(*p*, *T*_*C*_) represents the average frequency of phrase *p* in the current tweet set *T*_*C*_, and idf(*p*, *T*_*H*_) represents the inversed document frequency of the tweets with phrase *p* in the historical tweet set *T*_*H*_. The logarithm function is to scale up the fraction of rare tweets. The above algorithm is derived from the typical tf-idf algorithm [20]. The difference lies in the replacement of the largest word frequency in current tweet set with the size of current tweet set, which aims at eliminating the influence of other heat phrases. Then, PHI and SSI are calculated with the relative frequencies of all the phrases:

(6) PHI=∑p∈P1rfp,SSI=∑p∈P2rfp×orderp,

where *P*_1_ stands for the set of smog-related health hazard phrases, *P*_2_ stands for the set of smog severity phrases. Then, social network diffusion is considered to calculate D-PHI and D-SSI. We calculate the network diffusion-based average frequency:

(7) $$\begin{array}{c} daf\left(\;p,T_{C}\;\right)=\frac{\sum_{d\in T_{C}}^{}\left(f\left(p,d\right)\times \left(g\left(d\right)+1\right)\right)}{\left|T_{C}\right|+\sum_{d\in T_{C}}^{}g\left(d\right)}, \end{array}$$

where *g*(*d*) represents a tweet's total number of retweets and likes. Once daf is calculated, we use it to replace the average frequency af to calculate the relative frequency rf and further compute the value of D-PHI and D-SSI. Finally, the PHI, SSI, D-PHI, and D-SSI make up the feature vectors of social view in this problem.

Moreover, we also extract features from air quality, including both air pollution concentrations (CO, NO_2_, SO_2_, O_3_, PM_2.5_, and PM_10_) and air quality index (AQI) which comprehensively evaluates the air quality. We extract records from various meteorological elements, including humidity, cloud value, pressure, temperature, and wind speed, all of which have been proven to affect smog disasters greatly. For example, high wind speed and low cloud value usually leads smog pollution to decrease in the next day.

### 4.2. Optimal Retain Store Placement

The optimal placement of a retail store has been of prime importance. For example, a new restaurant set up in a street corner may attract lots of customers, but it may close months later if it is located in a few hundred meters down the road. In this paper, the optimal retain store placement problem is a rank problem. We calculate scores for each of the candidate areas and rank them. The top-ranked areas will be the optimal region for placing. We get the label data from Dianping score (http://www.dianping.com/) and assume that the data observed can evaluate the popularity of a place. For this problem, we analyze the data obtained and build social and physical view and then build a classifier according to our framework.

A strong regional economy usually indicates high demand according to recent studies [15, 21]. Therefore, we mined the block's neighbour's user reviews from http://www.dianping.com/.

*(1) Dianping Score.* For each region *g*, we will retrieve service quality, overall satisfaction, environment class, and consumption level by mining the reviews of business venues neighbourhood regions. Region *g* has *t* neighbourhoods. We access the users' opinions over the neighbourhood region *N*_*i*_ to form features.

*Overall Satisfaction.* Since the overall rating of a business venue in block *g* represents the satisfaction of users, we use the LBFS to get the overall ratings of all business venues located in *N*_*i*_ as a numeric score of overall satisfaction. Formally, we have

(8) $$\begin{array}{c} F_{s}=LBFS\left(\;\text{overall satisfaction},d,n,m\;\right). \end{array}$$

*Service Quality.* Similarly, we use the LBFS to compute the service rating of business venues in *N*_*i*_ and express the service quality of the neighbourhood as

(9) $$\begin{array}{c} F_{q}=LBFS\left(\;\text{Service Rating},d,n,m\;\right). \end{array}$$

*Environment Class.* The level of the surrounding environment can reflect the current environment class of the area. Therefore, we use LBFS to get environment rating as

(10) $$\begin{array}{c} F_{e}=LBFS\left(\;\text{environment rating},d,n,m\;\right). \end{array}$$

*Consumption Cost.* The average value of the consumer's behavior of the surrounding regions can reflect the current consumption of the area. So we use LBFS to get the consumption cost of business venues of neighbourhood as a feature:

(11) $$\begin{array}{c} F_{c}=LBFS\left(\;\text{average cost},d,n,m\;\right). \end{array}$$

Bus transits are slow and cheap and are mainly distributed in areas having a large number of IT and educational establishments. The price of real estate and the traffic congestion index indicate whether the facility planning is balanced. We exploit these features to uncover the implicit preferences for a neighbourhood.

*Bus-Related Features.* Medium income residents choose bus. Because most of the city's residents are from the background of the middle class, bus traffic may represent the bulk of the city's flow. We try to measure the arrival, departure, and the volume of the transition on the streets of each block. For a region *g*, we try to use BT as the set of bus trajectories of a city, each of which is denoted by a tuple 〈*p*, *d*〉, where *p* is a pickup bus stop and *d* is a drop-off bus stop [21].

*Bus Arriving, Departing, and Transition Volume.* We extract the arriving, departing, and transition volumes of buses from smart card transactions. Formally,

(12) FbBAV=p,d∈BT:p∉g,  d∈g,FbBLV=p,d∈BT:p∈g,  d∉g,FbBTV=p,d∈BT:p∈g,  d∈g.

*Density.* Recent studies have reported that price premiums of up to 10% are estimated for retail stores within 400 m of a large number of bus stops. The density of the bus station is positively correlated with the value of the retail store. Here, we use smart card transactions and propose alternative methods and strategies for density estimation of bus stop. In fact, the number of bus stops in a trip can be approximated by fare. Therefore, we calculate the distance to the ratio of the estimated density in the vicinity of the bus station.

(13) $$\begin{array}{c} F_{i}^{BSD}=\frac{\sum_{p\in g||d\in g}^{}dist\left(p,d\right)/fare\left(p,d\right)}{\left|\langle p,d\rangle \in BT:p\in g\text{, }d\in g\right|}. \end{array}$$

*Balance. *The balance of smart cards can show the pattern of consumption and supply behavior. If some residents always maintain a high balance on the smart card, the huge cost of bus travel may mean (1) these residents are more dependent on the bus, which shows the lack of subway and taxi nearby, and (2) these residents have to go to work in places far away. In other words, these places may be far away. So, we try to use the smart card balance as a feature:

(14) $$\begin{array}{c} F_{i}^{SCB}=\frac{\sum_{p\in g||d\in g}^{}balance\left(p,d\right)}{\left|\langle p,d\rangle \in BT:p\in g\text{, }d\in g\right|}. \end{array}$$

*Real Estate Features.* The real estate prices may reflect the purchasing power and economic index of this region. First, we collect the historical prices of each estate, and we use LBFS to calculate estate price of the neighbourhood of each block. Formally, we have

(15) $$\begin{array}{c} F_{i}^{RE}=LBFS\left(\;\text{estate price},d,n,m\;\right). \end{array}$$

*Traffic Index Features.* The flexibility of a region like convenient traffic may contribute to the popularity of a region. We obtain the traffic index from http://nitrafficindex.com/. Formally, we have

(16) $$\begin{array}{c} F_{i}^{TI}=LBFS\left(\;\text{traffic index},d,n,m\;\right). \end{array}$$

*Intraclass Competitiveness.* We measure the proportion of neighbouring places of the same type *γ*_*l*_ with respect to the total number of nearby places. Then, we rank areas in reverse order, assuming that the least competitive area is the most promising one:

(17) $$\begin{array}{c} F_{i}^{Intra}=-\frac{N_{\gamma l}\left(l,r\right)}{N\left(l,r\right)}. \end{array}$$

However, it is worth noting that the retail industry's competitive stores and marketing can have a positive or negative impact. For example, one would expect to place a bar in a region with plenty of nightlife locations, because there already exists a service system, and there are a lot of people attracted to the area. However, being surrounded by competitors also means to share customers.

*Interclass Competitiveness.* In order to consider the iterations between different place categories, we adopt the metrics defined by Jensen [22]. In practice, we use the intercategory coefficients described to weigh the desirability of the places observed in the area around the object; that is, the greater the number of the places in the area that attracts the object, the better the quality of the location. More formally, we define the quality of location for a venue of type *γ*_*l*_ as

(18) $$\begin{array}{c} F_{i}^{Inter}=\underset{\gamma_{p}\in \Gamma }{\sum }\log\left(\;\chi_{\gamma_{p},\gamma_{l}}\;\right)\times \left(\;N_{\gamma_{p}\left(l,r\right)}-\bar{N_{\gamma_{p}\left(l,r\right)}}\;\right), \end{array}$$

where $\bar{N_{\gamma_{p}\left(l,r\right)}}$ means how many venues of type *γ*_*p*_ are observed on average around the places of type *γ*_*l*_, Γ is the set of place types, and *χ*_*γ*_*p*_,*γ*_*l*__ are the intertype attractiveness coefficients. Formally, we get

(19) $$\begin{array}{c} \chi_{\gamma_{p}->\gamma_{l}}=\frac{N-N_{\gamma_{p}}}{N_{\gamma_{p}}\times N_{\gamma_{l}}}\sum_{p}\frac{N_{\gamma_{l}}\left(p,r\right)}{N\left(p,r\right)-N_{\gamma_{p}}}. \end{array}$$

*POIs.* The POIs indicate the latent patterns of this region which may have contributed to the placement. Moreover, the category of a POI may have a causal relation to it. Let *♯*(*i*, *c*) denote the number of POIs of category *c* ∈ *C* located in *g*_*i*_, and let *♯*(*i*) be the total number of POIs of all categories located in *g*_*i*_. The entropy is defined as

(20) $$\begin{array}{c} F_{i}^{POI}=-\underset{c\in C}{\sum }\frac{♯\left(i,c\right)}{♯\left(i\right)}\times \log\frac{♯\left(i,c\right)}{♯\left(i\right)}. \end{array}$$

*Business Areas.* Business areas have important influence on optimal placement. The locations will get more customers and resources in the business areas. We retrieve the geographic information of business areas from Baidu map api. Finally we build a feature related to business areas. Formally, we have

(21) Fd1=LBFSis neighbour business areas,d,n,m,Fd2=1if gi∈Business Area0if gi∉Business Area,Fd=Fd1,Fd2,

where “is_neighbour_business_areas” is a Boolean value which means whether the block's neighbour is a business area.

## 5. Experiments

### 5.1. Urban Data

Table 1 lists the data sources. For social view, we crawl online business reviews from http://www.dianping.com/, which is a site for reviewing business establishments in China. Each review includes the shop ID, name, address, latitude, longitude, consumption cost, star rating, POI category, city, environment, service, overall ratings, and comments. We also crawl huge data from Weibo (a twitter-like website in China) and Twitter with geotags. For physical view, we extract features from smart card transactions from open web API. Moreover, we crawl the traffic index from http://www.nitrafficindex.com/, which is open to the public. Finally, we crawl the estate data from http://www.soufun.com/, which is the largest real estate online system in China. Moreover, we crawl huge air quality data from http://www.pm25.in/. We collected POI and business areas data from Baidu maps for each city. The road network data was gathered from OpenStreetMaps. We collected meteorological data from the National Oceanic and Atmospheric Administration's (NOAA) web service and http://forecast.io/ every hour. All the experiments can be obtained from open web service. The source code of this paper can be obtained from https://github.com/zxlzr/ELM-for-Urban-Computing.

### 5.2. Metrics

In total, we have two main metrics for our framework, the precision and efficiency. For specific case studies, for smog-related health hazard prediction, we have the following.

*Precision and Recall.* The precision and recall are given as follows:

(22) Precision=∩prediction set, reference setprediction set,Recall=∩prediction set, reference setreference set.

For optimal retain store placement, we have the following.

*Normalized Discounted Cumulative Gain.* The discounted cumulative gain (DCG@*N*) is given by

(23) $$\begin{array}{c} DCG\left[\;n\;\right]=\left\{\begin{array}{cc} {rel}_{1} & \text{if }n=1\\ DCG\left[n-1\right]+\frac{{rel}_{n}}{\log_{2}n} & \text{if }n\geq 2. \end{array}\right. \end{array}$$

Later, given the ideal discounted cumulative gain DCG′, NDCG at the *n*th position can be computed as NDCG[*n*] = DCG[*n*]/DCG′[*n*]. The larger the value of NDCG@*N*, the higher the top-*N* ranking accuracy.

*Precision and Recall.* We choose to use a four-level rating system as (3 > 2 > 1 > 0). To simplify our evaluation task, we treat the ratings less than 2 as low values and ratings of 3 as high values. In a top-*N* block list *E*_*N*_ sorted in descending order of the prediction values, we define the precision and recall as precision@*N* = (*E*_*N*_∩*E*_≥2_)/*N* and recall@*N* = (*E*_*N*_∩*E*_≥2_)/*E*_≥2_, where *E*_≥2_ are blocks whose ratings are greater than or equal to 2. For efficiency, we recorded the training time and compare with baselines.

### 5.3. Results

For smog-related health hazard prediction, the stacked ELM is trained hidden layer ANN with 10 to 40 hidden nodes; the BP is trained ANN with 2 to 3 hidden layers and 8 to 15 nodes in each hidden layer. Two classic SVM regression methods, nu-SVR and epsilon-SVR, are provided by LIBSVM [23]. Random forest regression method is provided by sklearn. Meanwhile, The accuracies of the health hazard prediction models using our framework, nu-SVR, epsilon-SVR, and random forest, are shown in Table 2. *p*(nf) means the precision of methods without knowledge fusion. We can find that two ANNs' methods outperform the SVM regression methods and the random forest regression method in forecasting the next day PHI, and the ELM achieves slightly higher prediction accuracy than the multiple hidden layers ANNs trained by BP.

For optimal retain store placement, the models trained with a single city's data are used as baselines. Our datasets have the data obtained from five cities in China. The blocks in each city have a label {0,1, 2,3} from http://www.dianping.com/ based on the values observed by users. For example, we have “2_1_31.” “2” means the id of city, “13” means the id of block, and the final “2” is the label. We treat each city as a single domain, containing hundreds of blocks. For example, if we want to do optimal retain store placement in Hangzhou, we use 75% of data in Hangzhou and all the other cities' data to train. The remaining 25% of data in Hangzhou are for testing. Actually, Hangzhou is treated as target domain, while the other cities are source domains.

**Figure 4:**
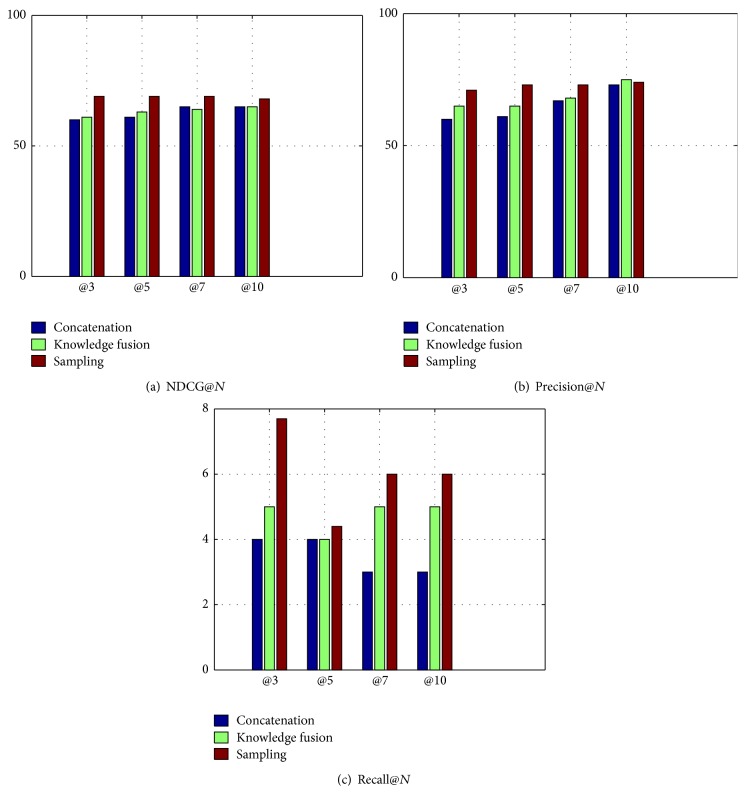
NDCG, precision, and recall of @*N* for Starbucks in Beijing.

Figure 4 shows the results of concatenation, knowledge fusion, and sampling methods for Starbucks. The concatenation method only concatenates social view and physical view into one single view to adapt to the learning setting. The results show that knowledge fusion works better. The sampling method means we manually filter some negative samples based on knowledge fusion, which will make better results as figure shows.

**Figure 5:**
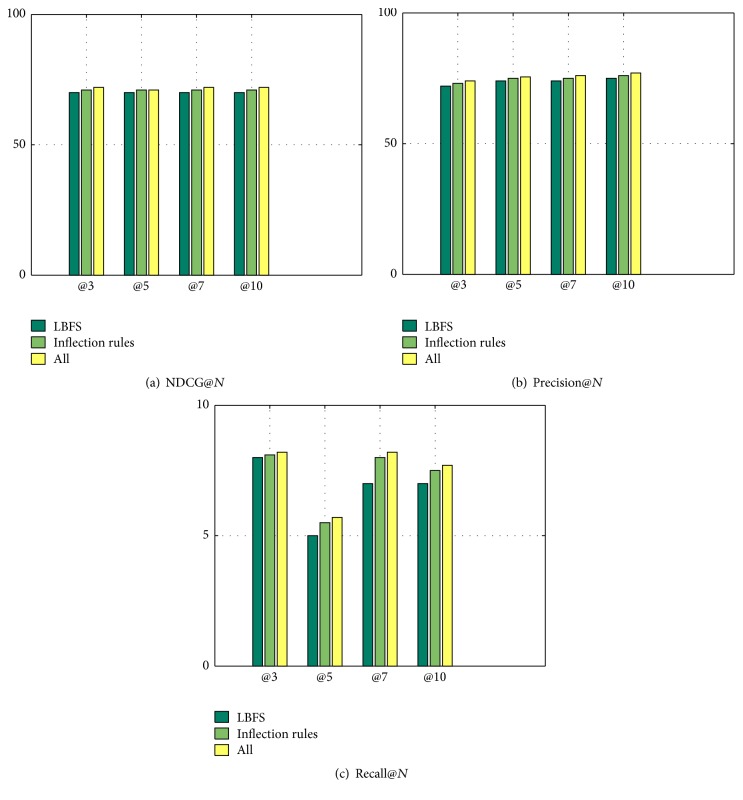
NDCG, precision, and recall of @*N* for Starbucks in Beijing.

Figure 5 shows the results of LBFS, inflection rules, and all methods for Starbucks. The LBFS method means we use location based feature selection to build features while not using the average value based on the methods in the past paragraph. Actually, LBFS works better. The inflection rules method means we use rules to calculate the final score if the area has spatial news such as “new subway, demolition.” The inflection rules consider the situations that our algorithm may not cover and make our method more robust. The all method includes DAELM, inflection rules, LBFS, sampling, and knowledge fusion. There is no doubt that the all method works best. Moreover, our framework performs more efficiently. In Table 3, we present the results obtained for the NDCG@10 metric and training time for all features across the three chains. The numbers in the brackets are minutes of training. In all cases, we observe a significant improvement in precision and efficiency with respect to the baseline.

## 6. Conclusion

In this paper, we propose a general application framework of ELM for urban computing and list three case studies. Experimental results showed that our approach is applicable and efficient compared with baselines.

In the future, we plan to apply our approach to more applications. Moreover, we would like to study the distribution of our framework so it can handle more massive data.

## Figures and Tables

**Table 1:** Details of the datasets.

Datasets	Size (M)	Sources
Comments	2,523	http://dianping.com/
Tweets	11,023	http://weibo.com/, http://twitter.com/
Buses	254	http://chelaile.net.cn/
Traffic	119	http://www.nitrafficindex.com/
Real estate	35	http://www.soufun.com/
Air	534	http://www.pm25.in/
POI, business areas	10	http://map.baidu.com/
Road network	9	http://openstreetmap.org/
Meteorological	98	http://forecast.io/, http://noaa.gov/

**Table 2:** The results of smog-related health hazard prediction.

Cities	Stacked ELM			BP			nu-SVR			Epsilon-SVR			Random forest		
	*p*(nf)	*p*	Time	*p*(nf)	*p*	Time	*p*(nf)	*p*	Time	*p*(nf)	*p*	Time	*p*(nf)	*p*	Time
Beijing	0.65	**0.80**	**3**	0.66	0.79	15	0.78	0.69	9	0.56	0.53	9	0.10	0.15	7
Tianjin	0.92	**0.89**	**3**	0.56	0.59	13	0.58	0.73	10	0.66	0.73	11	0.15	0.14	8
Shanghai	0.53	**0.88**	**4**	0.55	0.69	16	0.55	0.64	11	0.53	0.53	10	0.12	0.30	8
Hangzhou	0.31	**0.86**	**3**	0.63	0.65	10	0.54	0.55	9	0.66	0.83	9	0.15	0.34	7
Guangzhou	0.53	0.54	**5**	0.65	0.69	15	0.53	0.66	11	0.48	0.53	21	0.10	0.34	8
Average	0.75	0.73	**4**	0.56	0.56	15	0.59	0.65	10	0.59	0.73	10	0.20	0.34	7

**Table 3:** The best average NDCG@10 results of optimal retain store placement.

Cities	Starbucks	TrueKungFu	YongheKing
*MART (single city)*			
Beijing	0.743 (15)	0.643 (14)	0.725 (14)
Shanghai	0.712 (15)	0.689 (14)	0.712 (12)
Hangzhou	0.576 (13)	0.611 (12)	0.691 (10)
Guangzhou	0.783 (15)	0.691 (13)	0.721 (12)
Shenzhen	0.781 (16)	0.711 (15)	0.722 (13)

*RankBoost (single city)*			
Beijing	0.752 (23)	0.678 (20)	0.712 (19)
Shanghai	0.725 (25)	0.667 (20)	0.783 (21)
Hangzhou	0.723 (21)	0.575 (19)	0.724 (15)
Guangzhou	0.812 (22)	0.782 (19)	0.812 (18)
Shenzhen	0.724 (22)	0.784 (17)	0.712 (12)

*BP (Single city)*			
Beijing	0.753 (45)	0.658 (40)	0.702 (41)
Shanghai	0.724 (44)	0.657 (42)	0.7283 (44)
Hangzhou	0.725 (41)	0.555 (40)	0.714 (45)
Guangzhou	0.832 (45)	0.772 (42)	0.512 (41)
Shenzhen	0.722 (47)	0.754 (42)	0.702 (45)

*DAELM*			
Beijing	**0.755 (3)**	**0.712 (2)**	**0.755 (2)**
Shanghai	**0.745 (5)**	**0.751 (3)**	0.783 (1)
Hangzhou	**0.755 (5)**	**0.711 (2)**	**0.752 (5)**
Guangzhou	0.810 (9)	**0.810 (5)**	**0.823 (4)**
Shenzhen	0.780 (9)	0.783 (5)	**0.723 (8)**
